# Chemical Strategies for the Detection and Elimination of Senescent Cells

**DOI:** 10.1021/acs.accounts.3c00794

**Published:** 2024-04-11

**Authors:** Jessie García-Fleitas, Alba García-Fernández, Vicente Martí-Centelles, Félix Sancenón, Andrea Bernardos, Ramón Martínez-Máñez

**Affiliations:** † Instituto Interuniversitario de Investigación de Reconocimiento Molecular y Desarrollo Tecnológico (IDM), Universitat Politècnica de València, Universitat de València, Camino de Vera s/n, 46022 València, Spain; ‡ CIBER de Bioingeniería, Biomateriales y Nanomedicina (CIBER-BBN), Instituto de Salud Carlos III, 28029 Madrid, Spain; § Unidad Mixta UPV-CIPF de Investigación en Mecanismos de Enfermedades y Nanomedicina, Universitat Politècnica de València, Centro de Investigación Príncipe Felipe, C/Eduardo Primo Yúfera 3, 46100 Valencia, Spain; ∥ Unidad Mixta de Investigación en Nanomedicina y Sensores, Universitat Politècnica de València, Instituto de Investigación Sanitaria La Fe, Av Fernando Abril Martorell 106, 46026 Valencia, Spain; ⊥ Departamento de Química, Universitat Politècnica de València, Camino de Vera s/n, 46022 València, Spain

## Abstract

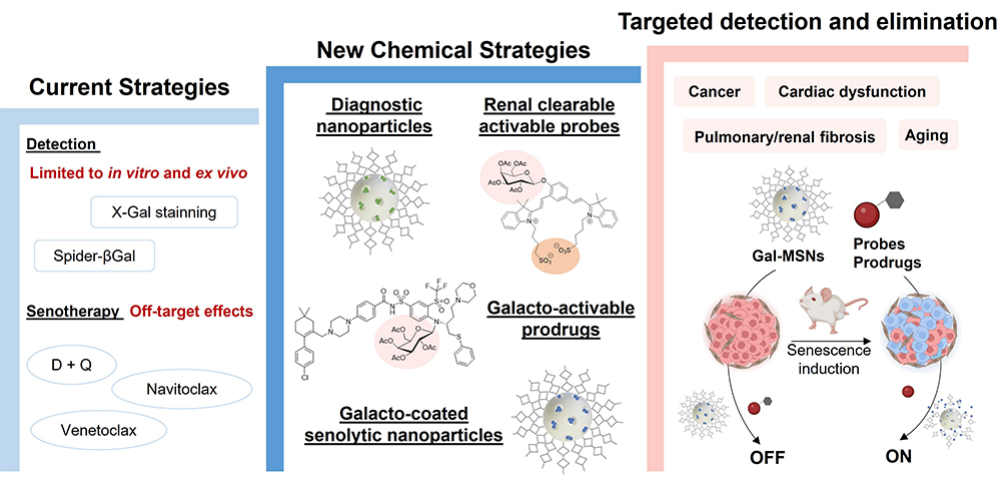

Cellular senescence can be defined as an irreversible stopping of cell proliferation that arises in response to various stress signals. Cellular senescence is involved in diverse physiological and pathological processes in different tissues, exerting effects on processes as differentiated as embryogenesis, tissue repair and remodeling, cancer, aging, and tissue fibrosis. In addition, the development of some pathologies, aging, cancer, and other age-related diseases has been related to senescent cell accumulation. Due to the complexity of the senescence phenotype, targeting senescent cells is not trivial, is challenging, and is especially relevant for *in vivo* detection in age-related diseases and tissue samples. Despite the elimination of senescent cells (senolysis) using specific drugs (senolytics) that have been shown to be effective in numerous preclinical disease models, the clinical translation is still limited due to the off-target effects of current senolytics and associated toxicities. Therefore, the development of new chemical strategies aimed at detecting and eliminating senescent cells for the prevention and selective treatment of senescence-associated diseases is of great interest. Such strategies not only will contribute to a deeper understanding of this rapidly evolving field but also will delineate and inspire new possibilities for future research.

In this Account, we report our recent research in the development of new chemical approaches for the detection and elimination of senescent cells based on new probes, nanoparticles, and prodrugs. The designed systems take advantage of the over-representation in senescent cells of certain biomarkers such as β-galactosidase and lipofuscin. One- and two-photon probes, for higher tissue penetration, have been developed. Moreover, we also present a renal clearable fluorogenic probe for the *in vivo* detection of the β-galactosidase activity, allowing for correlation with the senescent burden in living animals. Moreover, as an alternative to molecular-based probes, we also developed nanoparticles for senescence detection. Besides, we describe advances in new therapeutic agents to selectively eradicate senescent cells using β-galactosidase activity-sensitive gated nanoparticles loaded with cytotoxic or senolytic agents or new prodrugs aiming to increase the selectivity and reduction of off-target toxicities of current drugs. Moreover, new advances therapies have been applied *in vitro* and *in vivo*. Studies with the probes, nanoparticles, and prodrugs have been applied in several *in vitro* and *in vivo* models of cancer, fibrosis, aging, and drug-induced cardiotoxicity in which senescence plays an important role. We discuss the benefits of these chemical strategies toward the development of more specific and sophisticated probes, nanoparticles, and prodrugs targeting senescent cells.

## Key References

- Lozano-TorresB.; GalianaI.; RoviraM.; GarridoE.; ChaibS.; BernardosA.; Muñoz-EspínD.; SerranoM.; Martínez-MáñezR.; SancenonF.An OFF–ON Two-Photon Fluorescent Probe for Tracking Cell Senescence in Vivo. J. Am. Chem. Soc.2017, 139, 8808–881128625064 10.1021/jacs.7b04985.^1^*We designed here a naphthalimide-based two-photon probe for the detection of cell senescence that was validated in mice bearing tumor xenografts treated with senescence-inducing chemotherapy*.
- Lozano-TorresB.; BlandezJ. F.; GalianaI.; García-FernándezA.; AlfonsoM.; MarcosM. D.; OrzáezM.; SancenónF.; Martínez-MáñezR.Real-Time In Vivo Detection of Cellular Senescence through the Controlled Release of the NIR Fluorescent Dye Nile Blue. Angew. Chem., Int. Ed.2020, 59( (35), ), 15152–1515610.1002/anie.20200414232416002.^2^*We developed here mesoporous silica nanoparticles loaded with Nile blue and capped with a galacto-hexasaccharide for in vivo imaging of cellular senescence in palbociclib-treated BALB/cByJ mice bearing breast tumors*.
- Muñoz-EspínD.; RoviraM.; GalianaI.; GiménezC.; Lozano-TorresB.; Paez-RibesM.; LlanosS.; ChaibS.; Muñoz-MartínM.; UceroA. C.; GarauletG.; MuleroF.; DannS. G.; VanArsdaleT.; ShieldsD. J.; BernardosA.; MurguíaJ. R.; Martínez-MáñezR.; SerranoM.A Versatile Drug Delivery System Targeting Senescent Cells. EMBO Mol. Med.2018, 10, e935510.15252/emmm.20180935530012580 PMC6127887.^3^*We designed a versatile drug delivery system that was applied to the treatment of idiopathic pulmonary fibrosis in mice and tumors in in vivo models*.
- González-GualdaE.; Pàez-RibesM.; Lozano-TorresB.; MaciasD.; WilsonJ. R.; González-LópezC.; OuH. L.; Mirón-BarrosoS.; ZhangZ.; Lérida-VisoA.; BlandezJ. F.; BernardosA.; SancenónF.; RoviraM.; FrukL.; MartinsC. P.; SerranoM.; DohertyG. J.; Martínez-MáñezR.; Muñoz-EspínD.Galacto-Conjugation of Navitoclax as an Efficient Strategy to Increase Senolytic Specificity and Reduce Platelet Toxicity. Aging Cell2020, 19, e1314210.1111/acel.1314232233024 PMC7189993.^4^*We developed a galactose-conjugated derivative of Navitoclax that efficiently kills chemotherapy-induced senescent cells in xenografts and orthotopic in vivo models of non-small-cell lung cancer, resulting in impaired tumor progression, preventing platelet apoptosis in human samples and murine models*.

## 1 Introduction

Cellular senescence can be defined as an irreversible arrest of cell proliferation that arises in response to various stress signals^5^ including DNA-damaging agents, oxidative stress, mitochondrial dysfunction, oncogene activation, and exposure to cytotoxic drugs, among others.^6,7^ Apart from cell cycle arrest, senescent cells can also be defined by other hallmarks, such as the upregulation of pro-survival pathways, epigenetic changes, and their highly metabolically active state. This status includes the delivery of the so-called senescence-associated secretory phenotype (SASP) (a context-dependent and dynamic secretome).^8,9^ Senescent cells also exhibit a larger cell size and an increase in the lysosomal compartment, with the senescence-associated enzyme β-galactosidase (SA-β-Gal) being overexpressed.^6^

**Figure 1:**
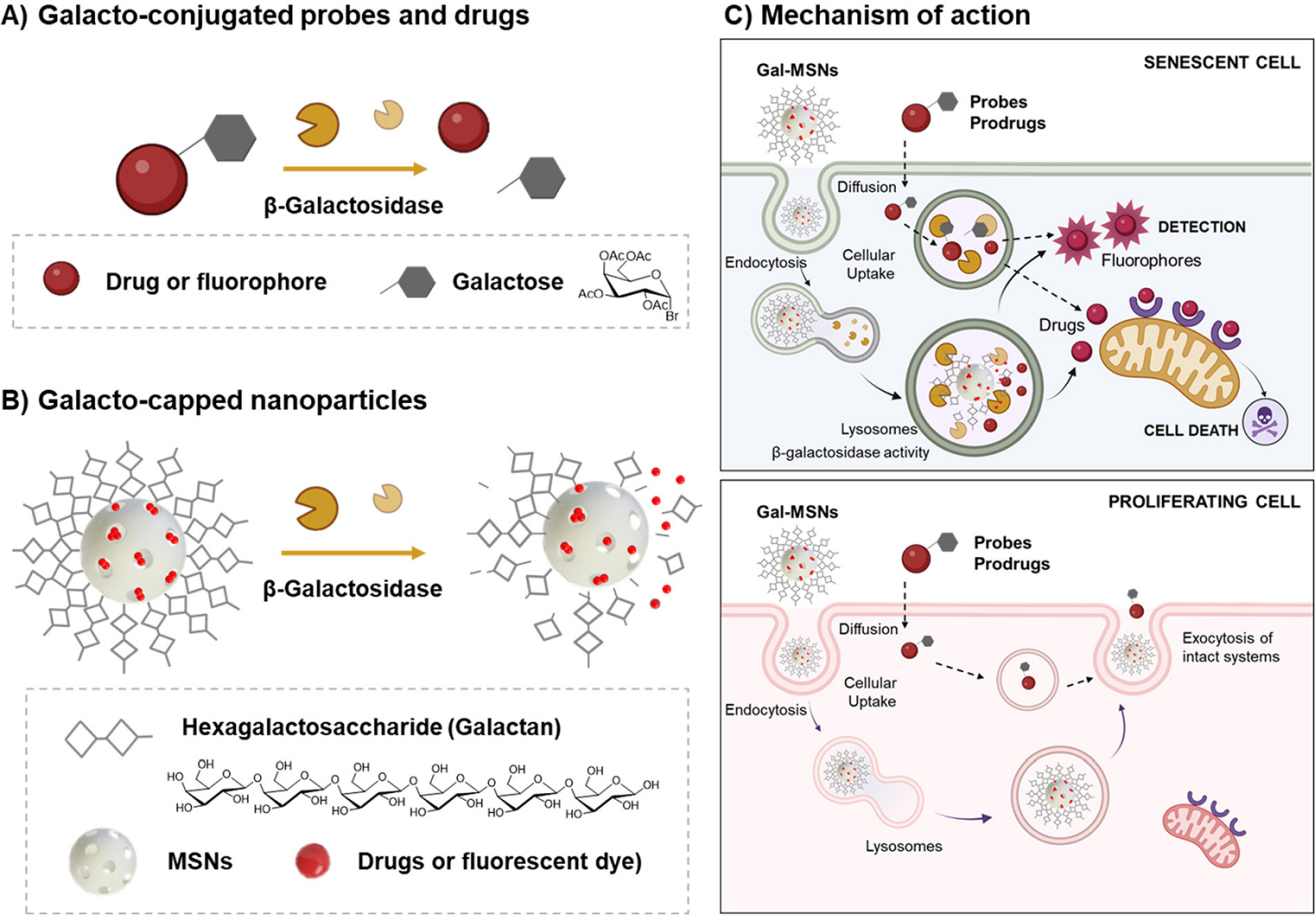
Representation of galacto-activable nanosystems for senescence cell detection and elimination. A) Molecular probes or drugs can be modified with a galactose moiety to obtain an inactive product. B) Mesoporous silica nanoparticles (MSN) are loaded with fluorophores or drugs, and the external surface is coated with the hexagalactosaccharide (galactan). C) These systems are triggered by the activity of senescence-associated β-galactosidase (overexpressed in senescent cells), where the enzyme catalyzes the hydrolysis of galactan into monosaccharides, while in proliferative cells they remain inactive.

The accumulation of senescent cells has been described both in physiological situations and in certain pathological processes in different tissues, exerting effects on processes as differentiated as embryogenesis, tissue repair and remodeling, cancer, aging or tissue fibrosis, and other age-related diseases.^10,11^ Besides, there is an accumulation of senescent cells with advancing age in the tissues that reflects the decline in cell repair mechanisms and in the capacity of the immune system to clear both damaged and senescent cells.^7,10−14^ In addition, senescent cells play a crucial role in the context of cancer by suppressing the proliferation of tumoral cells and stimulating immune clearance.^15^ However, senescent cells that become persistently present in tissues give rise to many pathological states, including cancer recurrence and other age-associated diseases such as atherosclerosis and other cardiovascular pathologies, idiopathic pulmonary fibrosis, etc. Within this context, senolysis,^16^ the study of the eradication of senescent cells selectively, is underway as a potentially encouraging intervention to treat diseases associated with aging or promote tissue rejuvenation. Besides, a crucial related issue is to have simple tools to monitor the presence of senescent cells. In this scenario, strategies for the detection and elimination of senescent cells are of importance. Based on this, we focus here on recent advances developed by our group in recent years that address these issues (detection and elimination of senescent cells) using molecule-based probes, nanoparticles, and prodrugs. In particular, we based our efforts on the development of different activable systems based on the β-galactosidase activity overexpressed in the lysosomes of senescent cells (Figure 1).

## 2 Detection of Cellular Senescence

Methods aiming to detect senescence markers are of importance; however, there is not a single universal marker, and the presence of senescent cells can be confirmed only by detecting multiple markers. The most noticeable macroscopic sign of senescence is a change in cell morphology, as senescent cells have a characteristically flattened and irregular appearance *in vitro*. Moreover, proteins responsible for cell proliferation, such as the tumor suppressors p53, p16Ink4a, and p21 that are activated during growth arrest, and a proliferation marker such as Ki67 and lower pRb levels have been widely used as suitable to detect senescent cells.^17,18^ Besides, cells in a state of replicative senescence typically exhibit shortened telomeres. Moreover, the delivery of a context-dependent dynamic secretome (SASP) is another hallmark of senescent cells. This SASP includes the secretion of different chemokines, cytokines, and proteases and all together are powerful markers of senescence.^19,20^ Other biomarkers, including lipofuscin, SA-β-Gal, and other lysosomal hydrolases (such as α-l-fucosidase, acid phosphatase, β-glucuronidase, β-hexosaminidase, *N*-acetyl-β-glucosaminidase, and α-mannosidase), are also overexpressed in senescent cells.^6^ In particular, acid β-galactosidase (encoded by GLB1) is the origin of SA-β-Gal activity due to the accumulation of acid β-galactosidase in the lysosomes of senescent cells.

Overexpression of β-galactosidase activity is perhaps one of the most extensively used and consolidated biomarkers of senescence.^21,22^ However, employing β-galactosidase activity as a senescence marker has the limitation that elevated activity of β-galactosidase is not exclusive to senescent cells as it is also observed in cells cultured under serum starvation or in confluent, quiescent cultured cells. Nevertheless, β-galactosidase is extensively used to detect senescent cells in culture and mammalian tissues using commercially available chromogenic/fluorogenic probes.^23−27^ Reported fluorescent molecular probes for β-galactosidase detection are mainly composed of two subunits: (i) a reactive fragment containing a galactose residue and (ii) a fluorophore as a signaling group. Usually, both subunits are covalently linked through an O-glycosidic bond (and the less common N-glycosidic bond) directly or through self-immolative fragments. The probes usually show low fluorescence, yet they became highly emissive after hydrolysis by β-galactosidase in senescent cells. Based on this, several probes have been described using coumarin, quinolines, fluorescein, naphthalimide, hemicyanines, benzothiazole, and dicyanomethylenepyran derivatives as signaling units.^1,3,28,29^

**Scheme 1:**
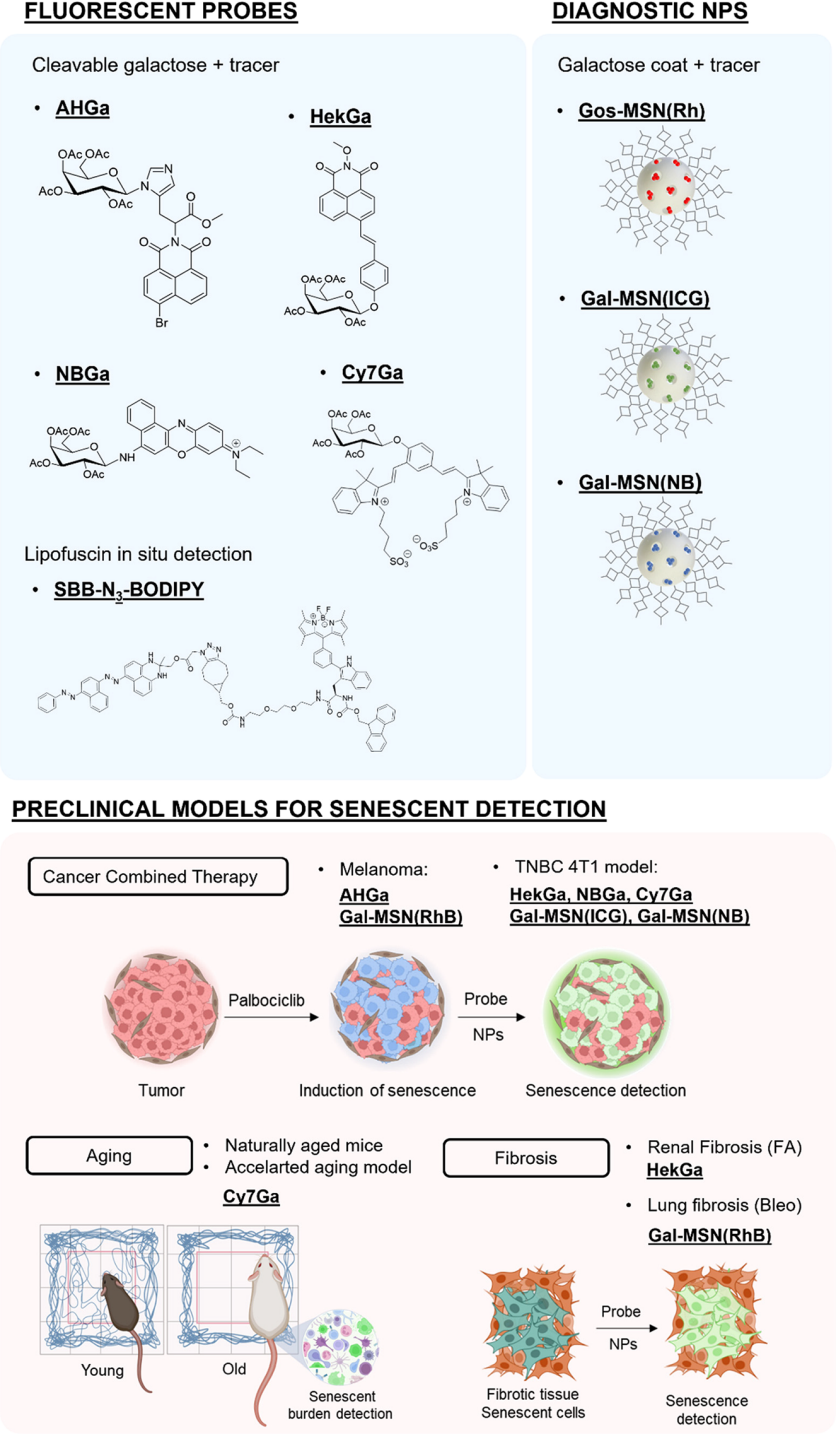
Summary of Chemical Strategies, Molecular Probes, and Nanoparticles, Developed to Detect Cellular Senescence in Different Scenarios

Despite major advances in recent years in the development of fluorescent molecules to detect β-galactosidase, some reported probes were synthesized using tedious multistep protocols, and they were usually assayed in *in vitro* or *in vivo* models not directly associated with senescence.^30−32^ Considering the aforementioned aspects, we contributed to this field by the development of new molecular probes or nanoparticles (Scheme 1).

**Figure 2:**
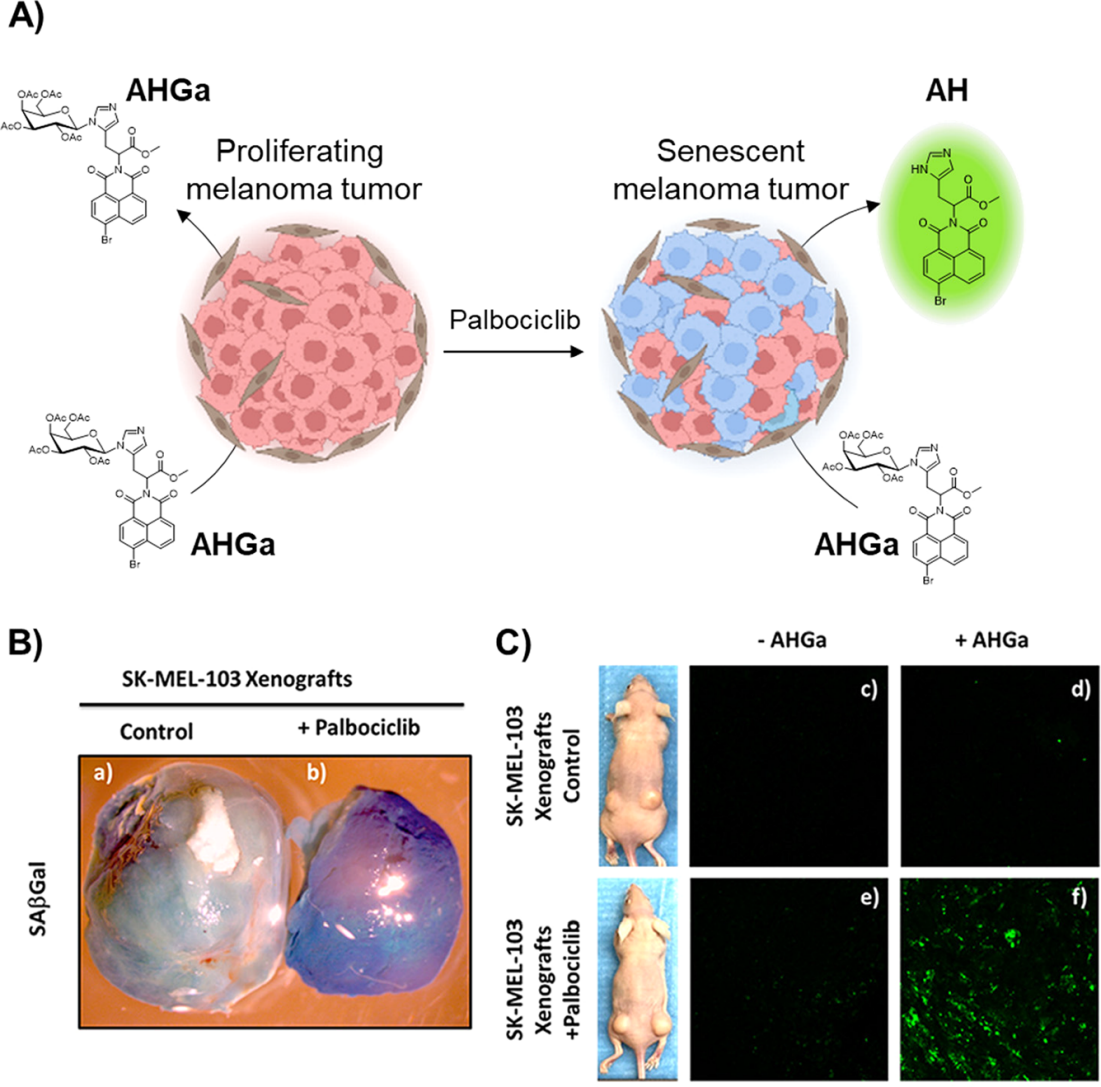
A) Activation of **AHGa** in senescent cells (schematic representation). B) Tumors of SK-Mel-103 a) control or b) SK-Mel-103 senescent tumors after SAβGal staining. C) Confocal images of SK-Mel-103 control cells from nonsenescent tumors c) vehicle, d) after injection of the **AHGa** probe; and SK-Mel-103 from senescent tumors e) vehicle, f) after injection of the **AHGa** probe. Adapted with permission from ref (1). Copyright 2017 American Chemical Society.

Our first contribution was in 2017 by designing a two-photon probe (**AHGa**) using a naphthalimide fluorophore (AH) as a signaling unit. **AHGa** is composed of an l-histidine methyl ester linker with an acetylated galactose, where one of the aromatic nitrogen atoms of l-histidine is bonded with an acetylated galactose via a hydrolyzable N-glycosidic bond. Furthermore, **AHGa** in PBS is poorly emissive (Φ_**AHGa**_ = 0.002), whereas an intense fluorescence is observed in the hydrolysis product (i.e., AH) (Φ_AH_ = 0.458, >200-fold enhancement) at 540 nm that remained unchanged in a wide pH range.^1^ Validation *in vitro* of **AHGa** was demonstrated in a senescent SK-Mel-103 (human melanoma) cell line, induced by palbociclib. By two-photon confocal microscopy (λ_exc_ = 750 nm), a remarkable increase (approximately 10-fold) in palbociclib-treated SK-Mel-103 cells was observed in the presence of **AHGa** compared to in control SK-Mel-103 cells (Figure 2A). The senescence-tracking ability of the **AHGa** probe was also corroborated *in vivo* using SK-Mel-103 tumor-bearing mice, where the senescence induction in the tumor was carried out by treatment with palbociclib (Figure 2B). The **AHGa** probe was administered intravenously via the tail vein, and after 3 h of treatment, the mice were sacrificed. Confocal microscopy *ex vivo* analysis corroborated that palbociclib-treated tumors in the presence of **AHGa** showed a strong fluorescence signal (approximately 15-fold) compared to that of control tumors (Figure 2C). This was one of the first OFF–ON two-photon fluorescent probes to detect senescence in a realistic *in vivo* model.

Based on a naphthalimide fluorophore, we developed the two-photon probe **HeckGa** for the *in vitro* and *in vivo* detection of cellular senescence.^33^**HeckGa** is based on a naphthalimide-styrene-fluorophore unit with an acetylated β-galactose covalently bonded to the anomeric carbon. **HeckGa** was assessed *in vitro* in senescent SK-Mel-103, A549 (human lung carcinoma), BJ (human fibroblast), and 4T1 (murine triple negative breast cancer) cell lines. The cell experiments showed a 3.6-fold fluorescence enhancement in the different senescent cellular models treated with **HeckGa** compared to nonsenescent cells under the same conditions. In this case, we validated the **HeckGa** probe *in vivo* in two different disease models of senescence: (a) C57BL/6 J male mice with renal fibrosis induced by folic acid (o.g.) and (b) BALB/cByJ female mice with 4T1 breast cancer tumors treated with palbociclib (o.g.). When BALB/cByJ were treated with palbociclib and injected intraperitoneally (i.p.) with **HeckGa**, *ex vivo* IVIS images showed a strong emission signal in senescent tumors (enhancement of ca. 4.6-fold) compared with proliferative tumors treated with the probe. Additionally, in the renal fibrosis model, mice injected with **HeckGa** exhibited a strong emission signal 5.8-fold higher in the kidneys than those from control mice (*ex vivo*).

Along the same lines, we have reported recently the development of a new senoprobe (**NBGa**) based on the conjugation of a galactose derivative with the near-infrared (NIR) fluorescent dye Nile blue (NB).^34^**NBGa** displays a low emission that increased markedly in the presence of human β-galactosidase enzyme (6.8-fold after 60 min) due to the hydrolysis of **NBGa** to give NB. The probe was validated *in vitro* in 4T1 and SK-Mel-103 cells incubated with palbociclib to induce senescence. Fluorescence quantification of confocal images confirmed an increased **NBGa** fluorescence emission (approximately 10-fold) in senescent cells compared with controls. The probe was also validated *in vivo* in female BALB/cByJ mice, which were orthotopically injected with 4T1 breast cancer cells for tumor development. The administration of **NBGa** (i.v.) in mice treated with palbociclib (to induce senescence in tumors) displayed *in vivo* (by IVIS) increases in fluorescence of ca. 4-fold at 0.5 h and 10-fold at 3 h in the tumor area. The **NBGa** probe constitutes a qualitative, rapid, and minimally invasive method for the direct detection of senescence *in vivo*.

**Figure 3:**
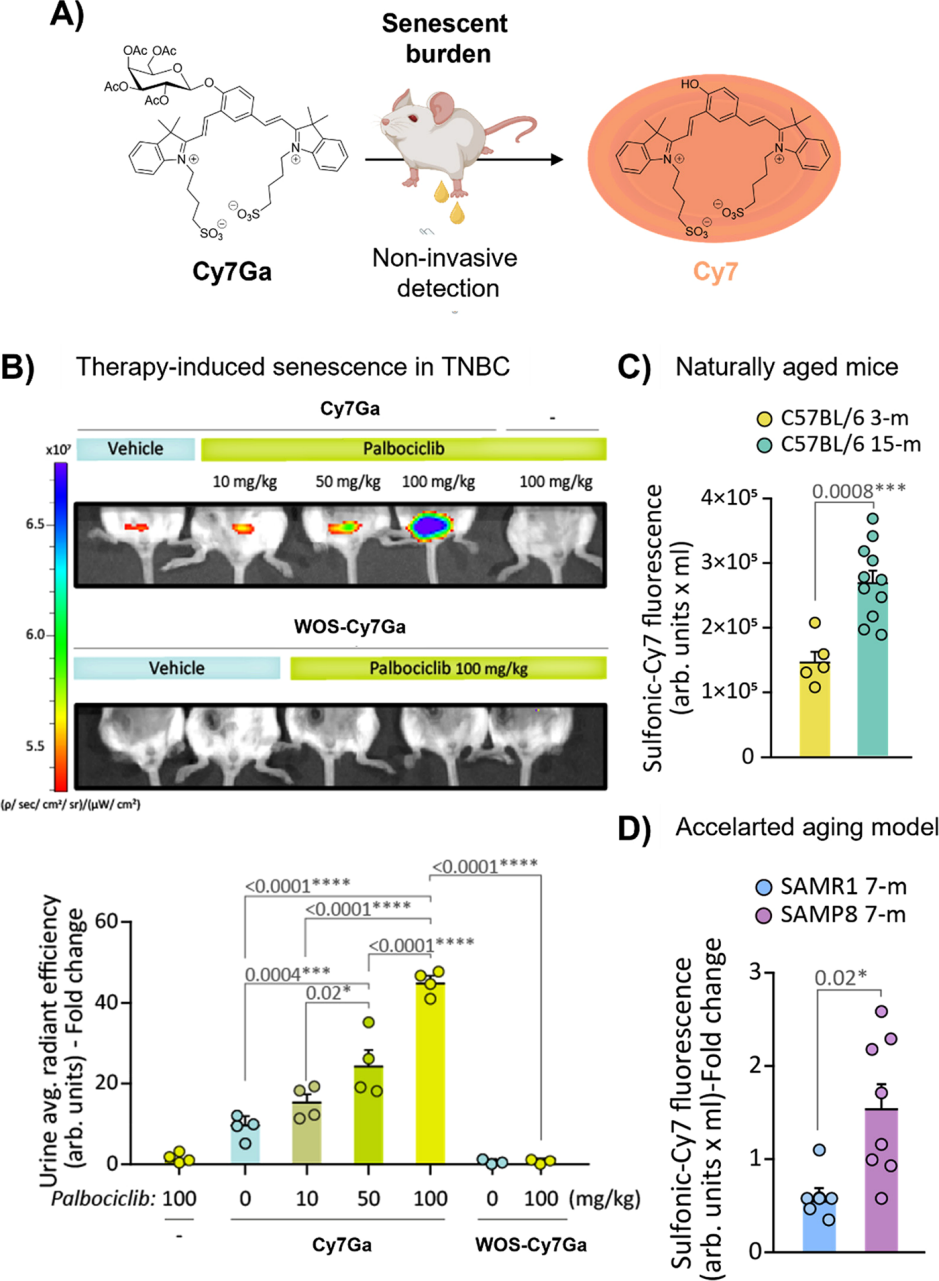
A) Representation of selectivity senescence detection and renal clearance of the **Cy7Ga** probe. B) At the top, *in vivo***Cy7Ga**-associated fluorescence IVIS imaging in bladders of BALB/cByJ female mice with 4T1 breast cancer tumors and treated orally with palbociclib (0, 10, 50, and 100 mg/kg), compared to mice treated with the highest dose of palbociclib but not injected with **Cy7Ga**. Using the same animal model injected with the **WOS-Cy7Ga** probe, we compared those receiving 100 mg/kg of palbociclib to mice not receiving any treatment. At the bottom, the IVIS readout of Cy7 average radiant efficiency in urine from mice. C) Cy7 urine fluorescence from 2- vs 14-m BALB/cByJ mice. D) Cy7 urine fluorescence of 7-m SAMP8 and SAMR1 mice. Adapted with permission from ref (35), with the Creative Commons CC BY license http://creativecommons.org/licenses/by/4.0/. Copyright 2024, the authors of the original publication.

These developed probes are fundamental tools for the detection of β-galactosidase enzymes in several disorders. Nevertheless, the small number of procedures to track β-galactosidase activity in live organisms is still a disadvantage limiting progress in this research area. In fact, the analysis of cellular senescence impact at the tissue level and its derived pathologies and in longitudinal studies to monitor senolytic treatments relies on our capacity to evaluate the presence of senescence in a simple and recurrent way. Besides, most of the available probes are mainly eliminated by the reticuloendothelial system, and this may result in the accumulation of these substances in the liver and spleen, potentially causing organ toxicity and side effects. An alternative to this is the design of renal-clearable probes, which is an area of growing interest. In this approach, we designed a **Cy7Ga** probe based on a Cy7 fluorophore containing sulfonic groups and attached to an acetylated galactose.^35^ The zwitterionic nature of the Cy7 fluorophore containing sulfonic groups enhances water solubility and also prevents serum protein binding, allowing rapid renal excretion.^36,37^**Cy7Ga** is poorly fluorescent (Φ_**Cy7Gal**_ = 0.0062, OFF state), yet hydrolysis with β-galactosidase results in the formation of highly fluorescent Cy7 (Φ_Cy7_ = 0.43, ON state), which is cleared by the kidneys, allowing its detection in the urine (Figure 3A). The probe was validated *in vivo* in the chemotherapy-induced senescence 4T1 cancer mouse model, treated with increasing concentrations of palbociclib (10, 50, or 100 mg/kg) to induce diverse levels of senescence burden within the tumors. In fact, a correlation between the increment of cellular senescence in tumors with the palbociclib dose was observed by immunohistochemical staining. The **Cy7Ga** probe was intraperitoneally (i.p.) administered, and after 15 min, IVIS images from mice *in vivo* displayed in the bladder a fluorescence accumulation, indicating rapid renal clearance of the Cy7 fluorophore. Then, after mice recovered from anesthesia, urine was collected, and we observed an increase in emission in the urine for mice treated with increasing quantities of palbociclib and therefore with the burden of cellular senescence in the tumors. We also demonstrated that renal clearance of Cy7 dye is due to the zwitterionic nature of the probe containing sulfonic groups (Figure 3B) as a similar probe lacking sulfonic groups (**WOS-Cy7Ga**) was not renally cleared. Finally, the ability of probe **Cy7Ga** to evaluate β-galactosidase activity during aging was validated in naturally aged BALB/cByJ (Figure 3C), SAMR1, and SAMP8 mice (Figure 3D). In all cases, the probe readout correlates with a higher senescence burden in old mice versus young animals. In addition, a good correlation between senescent cell increases and Cy7 emission in urine was observed in aged animals and during senolytic intervention. This is the first renal-clearable fluorogenic probe for the *in vivo* detection of β-galactosidase activity during senolysis and aging. Such renal-clearable probes can be the basis for the development of poorly invasive protocols for *in vivo* monitoring of senolytic therapeutic treatments and senescence detection.

**Figure 4:**
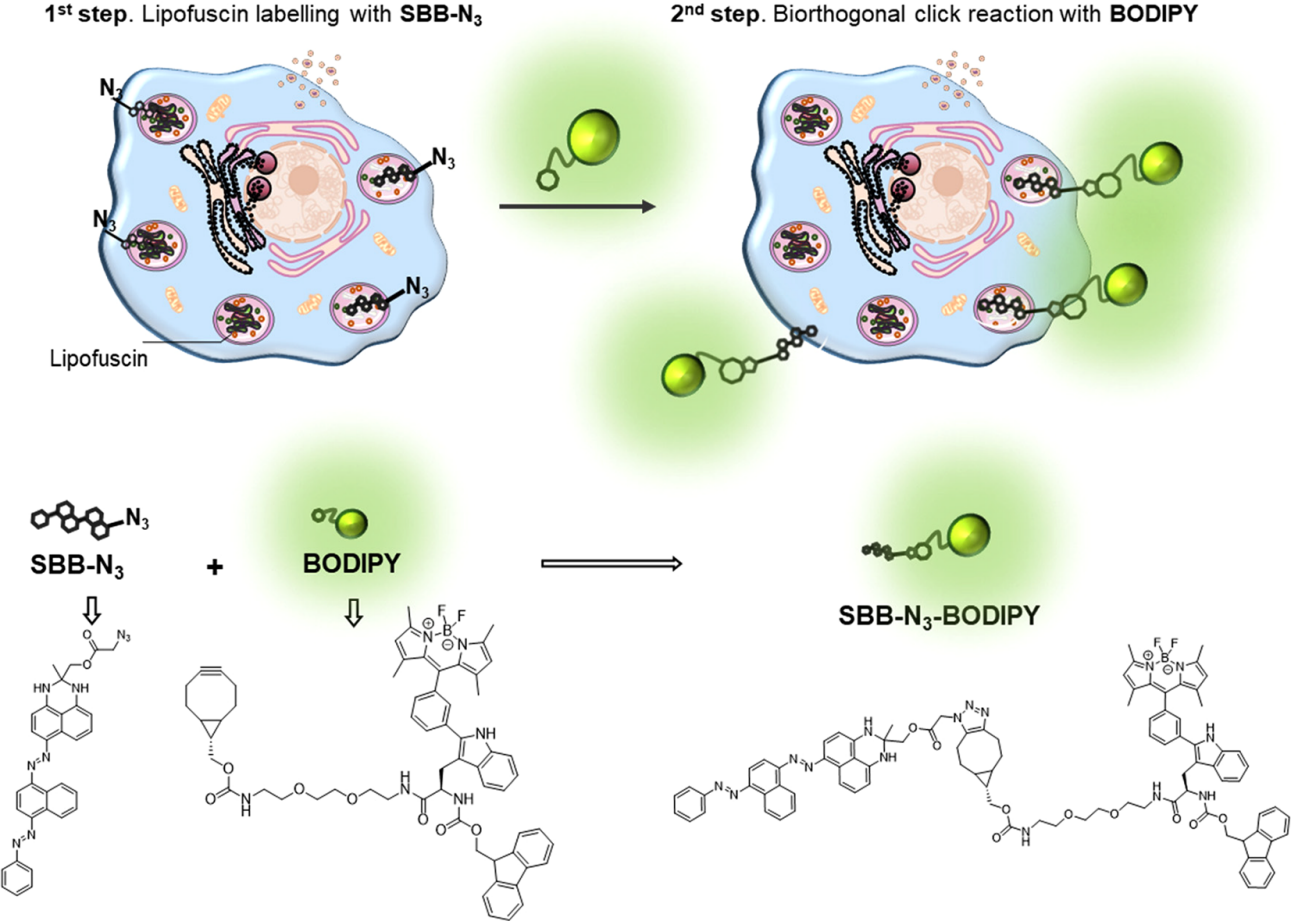
Detection of a senescent cell by binding lipofuscin to a Sudan Black B derivative containing an azide moiety (SBB-N3) and consequent reaction with a fluorophore having a cyclooctene ring in its structure (BODIPY). Adapted with permission from ref (38). Copyright 2023, John Wiley and Sons.

Alternatively, in an attempt to design probes not based on the overexpression of β-galactosidase enzyme in senescent cells, a two-step protocol through lipofuscin labeling with a fluorescent reporter was designed.^38^ The lipofuscin detection can be achieved using basic dyes (such as Sudan Black B (SBB)) that interact with the acidic groups of the lipids. This histochemical staining protocol is normally used as an auxiliary technique when SA-β-gal cannot be applied. While SA-β-gal staining requires fresh tissue, SBB staining is applicable in formalin-fixed and paraffin-embedded tissue sections. Based on this, we proposed a new method for the detection of senescent cells that involves the interaction of lipofuscin with a Sudan Black B derivative containing an azide moiety (SBB-N_3_) and the further addition and reaction with a fluorophore having a cyclooctene ring in its structure (BODIPY) (Figure 4). The effectiveness of the protocol was tested in drug-induced senescence cellular models (melanoma SK-Mel-103, TNBC MDA-MB-231, and WI-38 fibroblasts). A fluorescence pattern was observed in senescent cells (where the **SBB-N**_**3**_**-BODIPY** derivative was formed) but not in proliferative cells. This method provides an alternative tool for the detection of senescent cells based on an in situ bio-orthogonal reaction for lipofuscin labeling, which differs from the classical procedures based on the detection of the overexpression of lysosomal β-galactosidase activity. Overall, the probe allows the possible detection of senescence based on the lipofuscin marker by fluorescence in living cells. However, there are still some limitations for its application in tissue and living animals, compared to the galacto-activable probes able of detection and real-time monitoring of senescence.

As an alternative to fluorescent molecular probes, we also described organic–inorganic hybrid materials functionalized with molecular gates for senescence detection. These materials used mesoporous silica nanoparticles (MSNs) as an inorganic scaffold in which a fluorescent reporter is entrapped in its porous network. Besides, the external surface was functionalized with oligosaccharides containing several β-galactose units. The bulky galactooligosaccharides anchored onto the external surface of the nanoparticles inhibited reporter release, whereas in the presence of β-galactosidase a marked delivery is observed due to its enzymatic hydrolysis. These gated materials presented several advantages when compared to molecular fluorescent probes such as chemical stability, the possibility to integrate targeting ligands onto its external surface, and inherent amplification features because opening of the pores by enzymes induces the release of myriads of reporter molecules from the interior of the pores.^39^

Following these concepts, we published in 2012 the first example in which the selective detection of senescent cells using nanoparticles was described.^40^ The system used was based on MSNs loaded with rhodamine B (Rh) and capped with galacto-oligosaccharides (galacto-oligosaccharides of different lengths, Gos) which was previously reacted with 3-aminopropyl triethoxysilane (**Gos-MSN(Rh)**). Where the β-galactosidase enzyme is not present, a nearly zero delivery of the fluorophore was observed; however, as clear contrast, in the presence of the enzyme, rhodamine B is selectively released. The nanoparticles were validated *in vitro* in yeast cells overexpressing β-galactosidase enzyme in human fibroblasts of X-linked Dyskeratosis Congenita (X-DC1777 and X-DC4646) and in aged control human fibroblasts (DC7118). In all cases, a marked rhodamine B emission was found due to the overexpression of β-galactosidase enzyme compared to control cells.

**Figure 5:**
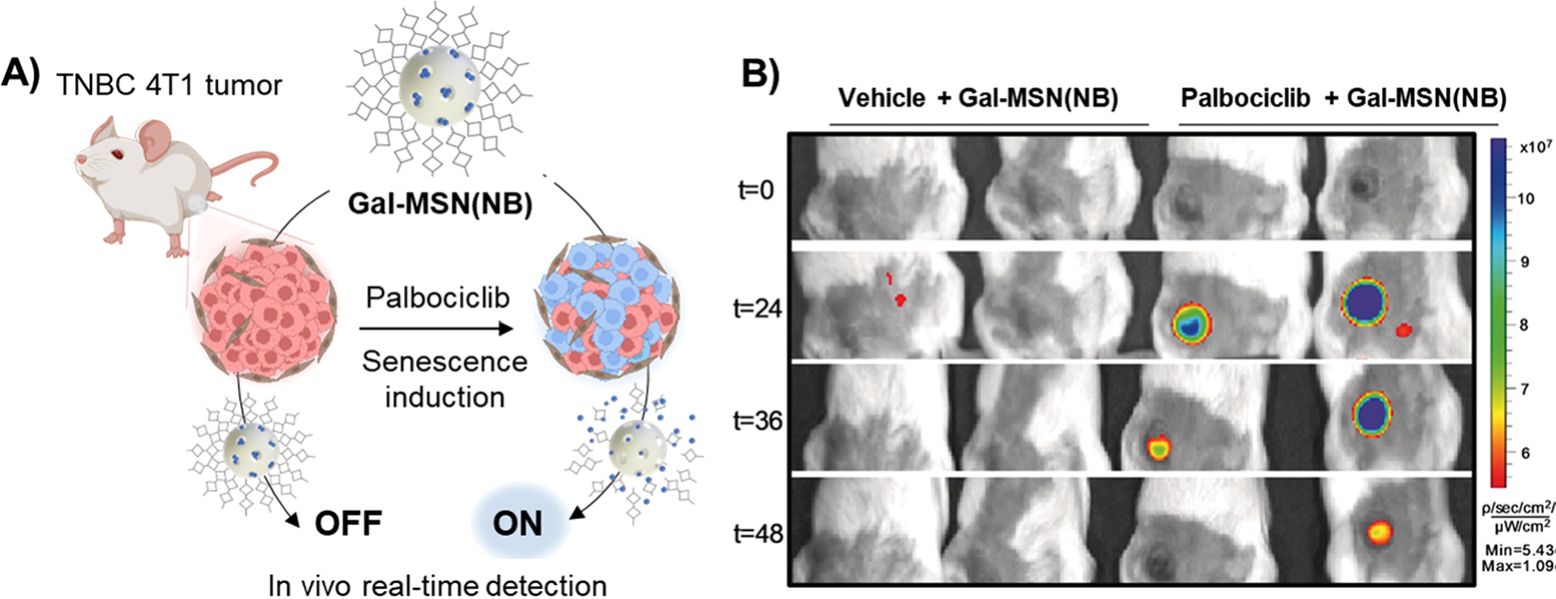
A) Representation of **Gal-MSN(NB)** activation in the 4T1 breast cancer tumoral model. After tumor formation, mice were treated with palbociclib (resulting in senescence tumors). Subsequently, they received **Gal-MSN(NB)** treatment, allowing *in vivo* detection of cellular senescence. B) *In vivo* imaging system (IVIS) captured images at various time points depicting BALB/cByJ female mice with 4T1 breast tumors. From left to right are control mice treated with **Gal-MSN(NB)** and mice treated with palbociclib and **Gal-MSN(NB)**. Adapted with permission from ref (2). Copyright 2020 Wiley-VCH.

In a continuation of these studies, we prepared similar nanoparticles loaded with NB fluorophore and capped with a commercially available β-(1,4)-hexagalacto-saccharide (**Gal-MSN(NB)**).^2^ NB forms poorly emissive π-stacked aggregates inside the pore voids, yet it is highly fluorescent when it is delivered from the MSN in the presence of the β-galactosidase enzyme. **Gal-MSN(NB)** was tested in palbociclib-sensitized SK-Mel-103 and 4T1 cancer cells. Confocal microscopy studies showed an intense fluorescent signal in senescent cells (7.0- and 10-fold for senescent SK-Mel-103 and 4T1 cells versus nonsenescent counterparts, respectively). *In vivo* detection of cellular senescence was carried out using BALB/cByJ female 4T1 tumor-bearing mice (Figure 5A). Mice treated with palbociclib (resulting in senescent tumors) and then with **Gal-MSN(NB)** (i.v.) showed a strong fluorescence signal in IVIS analysis in the tumors (4.3- and 7.3-fold enhancements after 24 and 36 h, respectively) when compared to mice treated only with the nanoparticles (Figure 5B). *Ex vivo* IVIS analysis of the organs of mice treated with palbociclib and **Gal-MSN(NB)** showed strong emission in the tumors which is 17.6-fold higher than that observed in the nonsenescent tumors or in other organs confirming the suitability of the nanoparticles for *in vivo* imaging of senescence.

## 3 Cellular Senescence Elimination as Therapy: Senotherapies

**Scheme 2:**
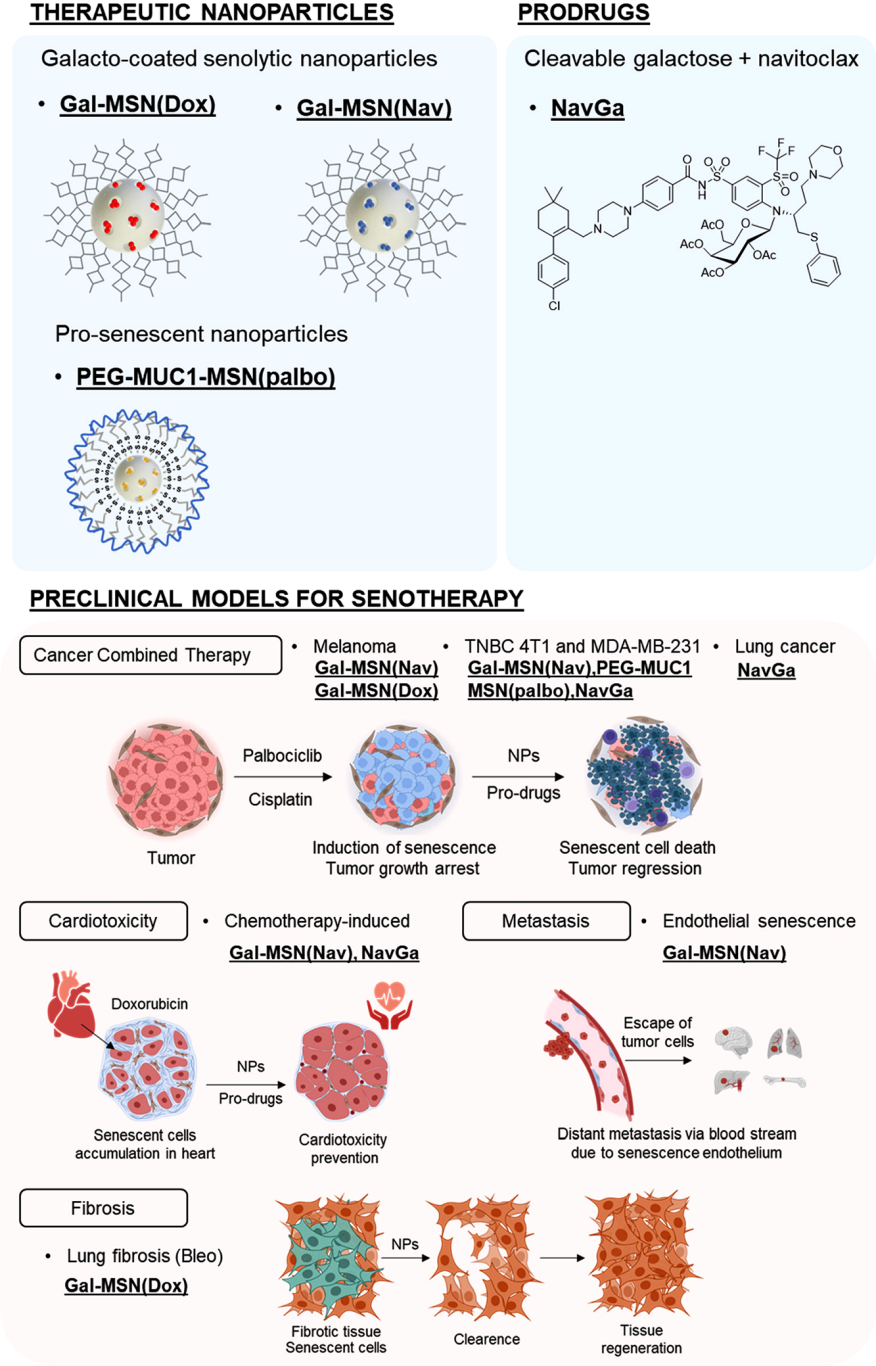
Summary of Chemical Strategies, Nanoparticles, and Prodrugs, Developed for Eliminating Senescent Cells in Different Preclinical Models of Disease

The elimination of senescent cells has raised hope for the prevention of multiple senescence-related disorders. In this scenario, the development of new therapies for the elimination of senescent cells (senotherapies) has recently raised attention. Senotherapies employ drugs to mitigate the deleterious effects of senescent cells through their elimination (senolytics), by inhibition of SASP (senomorphics), or by inhibition of senescence before it happens (senoblockers).^41^ Examples of senolytics are the combination of dasatinib and quercetin (D+Q), fisetin, navitoclax (ABT-263), ABT-737, a D-retro inverso (DRI)-isoform of FOXO4, HSP90 inhibitors, ATM inhibitors, the caspase-3 inhibitor piperlongumine, and cardiac glycosides, among others.^42−45^ These senolytics have been able to effectively reduce cancer relapse as well as delay the onset of aging-associated diseases and are already being evaluated in clinical trials. For instance, the senolytic combination of quercetin, a flavonoid that acts as a BCL-XL inhibitor, and dasatinib, an inhibitor of several tyrosine-kinases, has been studied in idiopathic pulmonary fibrosis in humans; however, the pilot study shown only limited efficacy.^46^ Besides, the clinical translation has been limited in most cases due to associated toxicities. The potent senolytic drug navitoclax (ABT-263), an inhibitor of the pro-survival BCL-2 family proteins BCL-2, BCL-XL, and BCL-W, causes dose-limiting thrombocytopenia.^47^ It was therefore imperative to identify the “second generation” of senolytic drugs. In this scenario, galacto-conjugation of senolytic drugs such as navitoclax, gentamicine, and duocarmycin has been explored.^48,49^ On the other side, nanoparticles targeting cell membrane receptors of senescent cells (CD9 or B2M) or with β-galactosidase-responsive coatings have been used for the delivery of senolytics.^50,51^ We have contributed to the field by designing some new senolytics based on nanoparticles or prodrugs to improve the efficacy and safety of current therapeutic approaches in several applications (Scheme 2).

In 2012, we first demonstrated the ability of **Gos-MSN(Rh)** nanoparticles (*vide ante*) to selectively deliver the cargo in senescent cells, which was inspired in previous nanoparticles capped with lactose that we previously reported in 2009.^52^ Based on a similar strategy, we developed in 2018 MSNs capped with a hexa-galacto-oligosaccharide (Gal) capable of releasing the payload when β-galactosidase is present. Targeting of senescent cells was demonstrated with nanoparticles loaded with the dye Rh (**Gal-MSN(Rh)**) or indocyanine green (ICG) **Gal-MSN(ICG)**, whereas the elimination of senescent cells was achieved with gated nanoparticles loaded with doxorubicin (**Gal-MSN(Dox)**) and senolytic drug navitoclax (**Gal-MSN(Nav)**).^3^ The nanoparticles were used in fibrosis and cancer models.

Nanoparticles were studied in tumor xenografts of NCI-H226 lung squamous carcinoma cells and SK-Mel-103 cells in mice treated with palbociclib. Remarkably, both **Gal-MSN(Dox or Nav)** demonstrated a clear therapeutic benefit after treatment with palbociclib. In contrast, the nanoparticles had no effect on tumor growth in the absence of palbociclib, demonstrating that their therapeutic activity requires the induction of senescence. Similar results were obtained with NCI-H226, where xenografts were treated with **Gal-MSN(Dox)**. It was also demonstrated that encapsulation reduced the toxic side effects of the drugs.^3^

**Figure 6:**
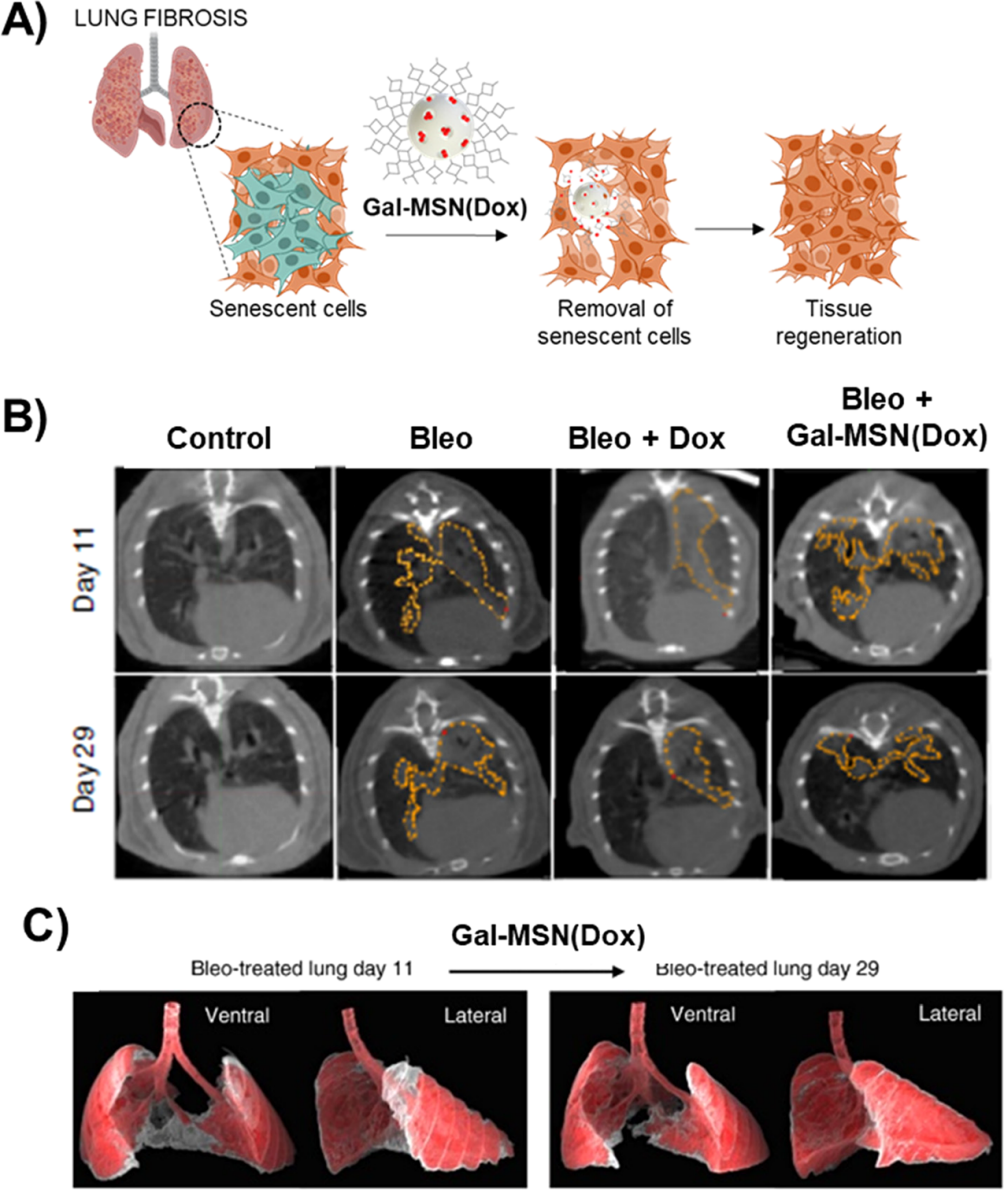
A) Representation of lung fibrosis therapy with **Gal-MSN(Dox)**. B) Representative images of computerized tomography of the indicated treatments at days 11 and 29 postbleomycin injury. Both images, at days 11 and 29, are for the same mouse. C) 3D isocontour-based volume rendering of a representative lung before and after treatment with **Gal-MSN(Dox)**. Fibrotic lesions are shown in gray, and healthy lung tissue is shown in red. Ventral and lateral views are shown. Adapted with permission from reference (3) with the Creative Commons CC BY license http://creativecommons.org/licenses/by/4.0/. Copyright 2018, the authors of the original publication.

The **Gal-MSN(Dox)** nanoparticles were also validated in a mouse model with idiopathic pulmonary fibrosis induced by bleomycin. In a period of 2 weeks, bleomycin intratracheal instillation in mice produced full-blown lung fibrosis, where cellular senescence is abundant (Figure 6A). Then, free doxorubicin or **Gal-MSN(Dox)** was administrated daily to bleomycin-treated mice. At the end of the treatment, only mice treated with **Gal-MSN(Dox)** presented LR/Cdyn (lung resistance and dynamic compliance) values similar to those of healthy controls, reducing collagen deposition and restoring pulmonary function. However, this behavior is not present in treatments with free doxorubicin (Figure 6B,C). Overall, these results corroborated the potential and versatility of nanoparticles in relevant senescence-associated human diseases.^3^

**Figure 7:**
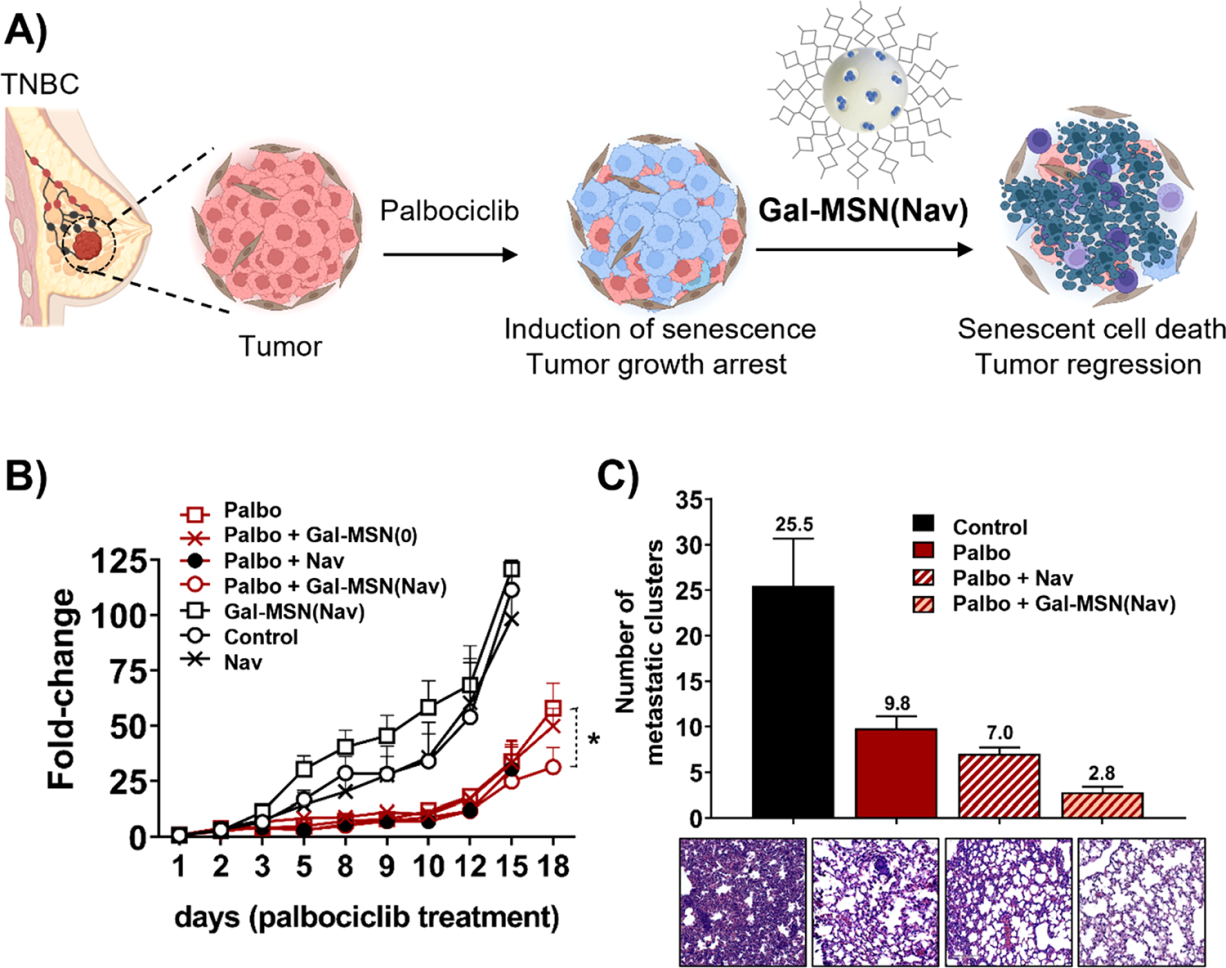
A) Representation of cancer combined therapy of palbociclib with **Gal-MSN(Nav)**. In TNBC tumors. B) The relative volume change of Balb/cByJ female mice orthotopically injected with 4T1 breast cancer cells and treated daily in comparison to its baseline before treatment. C) Quantification of lung metastasis and representative H&E lung sections. Adapted with permission from reference (53). Copyright 2020, Elsevier.

In an attempt to expand the potential use of nanoparticles applied to senolytic therapies and considering the effectiveness of drug-induced senescence in patients with locally advanced metastatic breast cancer,^11,15^ we also demonstrated that a combination of senescence induction and the elimination of senescent cells (using the **Gal-MSN(Nav)** nanoparticles) is an efficient means to inhibit tumor relapse in an aggressive triple-negative breast cancer (TNBC) subtype (Figure 7A).^53^ In this study, 4T1 cells were injected into the mammary pads of Balb/cByJ female mice (4–6 weeks) to induce tumor formation, and after 1 week of tumor growth, mice were treated with palbociclib. Further treatment with **Gal-MSN(Nav)** provided a therapeutic benefit when combined with palbociclib (Figure 7B); tumor-bearing mice treated in this manner survived to the experimental end point without significant changes in body weight, whereas treatment with palbociclib in combination with free navitoclax led to reduced survival and significant weight loss in surviving animals. Importantly, galactosidase activatable nanoparticles partially alleviated thrombocytopenia compared with the use of the free navitoclax. Additionally, TNBC model mice treated with palbociclib and **Gal-MSN(Nav)** present fewer lung metastases when compared to mice treated with palbociclib only, a remarkable result in terms of long-term prognosis and survival (Figure 7C).

In the same scenario, we investigated the role of endothelial senescence in cancer development and metastasis and the consequence of its elimination using targeted senolysis with **Gal-MSN(Nav)** nanoparticles.^54^ Initially we demonstrated that by the administration of 2 μM palbociclib it is possible to induce senescence in human umbilical vein endothelial cells (HUVEC). Moreover, we demonstrated that endothelial senescence promoted *in vitro* the migration of MDA-MB-231 breast cancer cells, and this effect was not observed with nonsenescent HUVEC cells. Focused on the impact of removing endothelial senescent cells, **Gal-MSN(Nav)** nanoparticles showed greater efficacy to eliminate senescent HUVEC cells compared with free navitoclax. The selective senescent HUVEC cell elimination also resulted in the restored functionality of endothelial tissue. Finally, **Gal-MSN(Nav)** and navitoclax were tested in the previously described 4T1 orthotopic TNBC mouse model (*vide ante*) to evaluate the effect of palbociclib-inducing senescence therapy followed by senolysis on the vascular endothelium. The study demonstrated that the induction of senescence in veins in the TNBC mouse model was induced by palbociclib systemic treatment and favors cancer cell migration, indicated by an increase in lung metastases. In contrast, animals treated with the senolytic therapy did not present vascular senescence, which also correlates to a reduction of metastatic burden.

**Figure 8:**
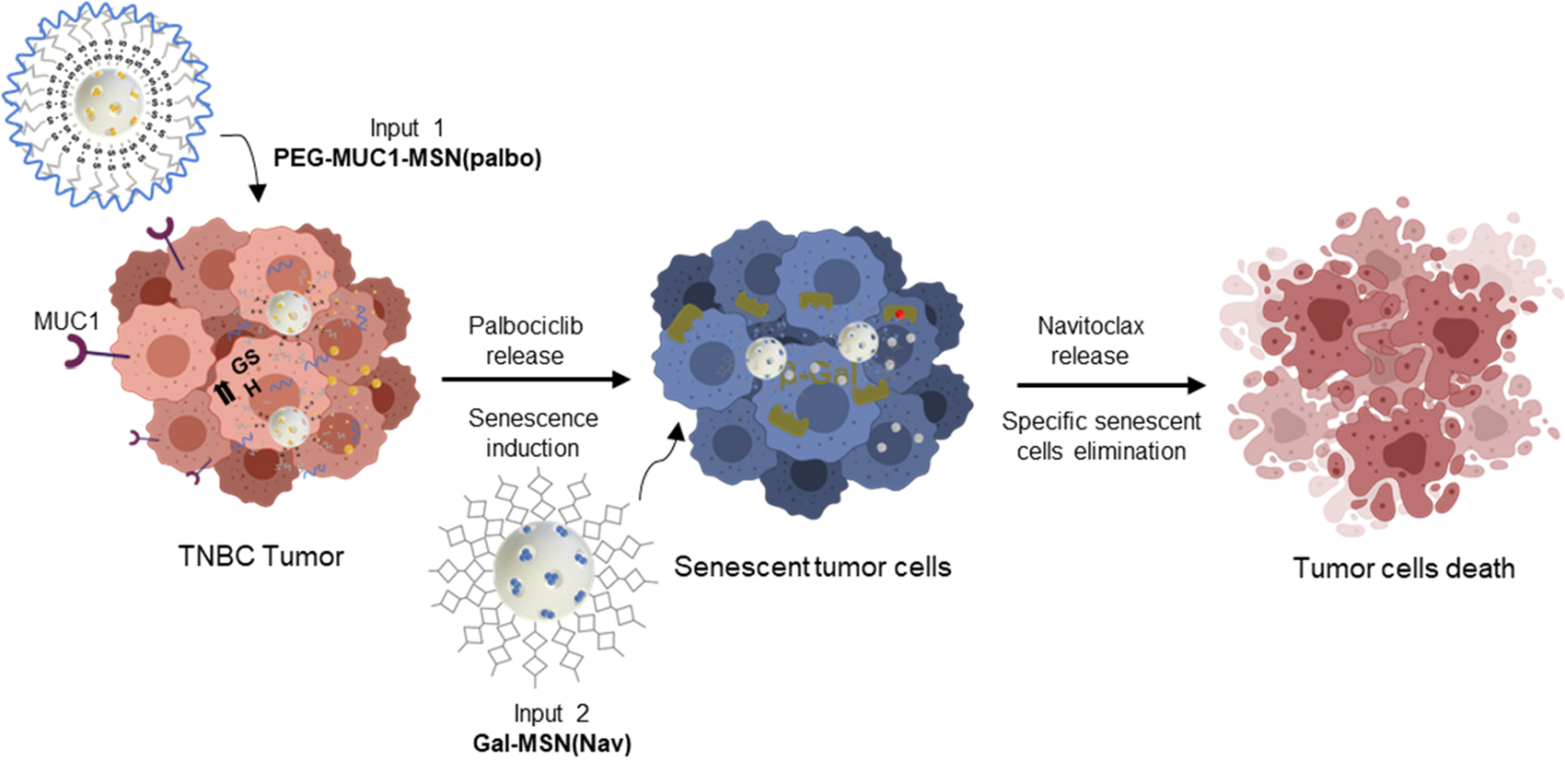
Representation of nanoparticle communication through stigmergy for improved tumor reduction via targeted induction of senescence and senolysis. The first community of nanoparticles (**PEG-MUC1-MSN(palbo)**) releases palbociclib, altering the environment through the induction of senescence in tumor cells. This enables a second community of nanoparticles (**NP(nav)-Gal**) to release their content (navitoclax) into senescent cells. This eliminates senescent tumor cells through apoptosis, resulting in a reduction in the tumor size. Adapted with permission from reference (55). Copyright 2023, Elsevier.

The research described above highlights the potential of the targeted induction of senescence followed by targeted senolysis to mitigate cancer progression and metastasis. Following the one-two-punch strategy, we combined two different nanoparticles for cancer treatment. We designed a first community of nanoparticles based on MSNs encapsulated with palbociclib and coated with a heterobifunctional poly(ethylene glycol) containing a disulfide bond covalently bonded to a MUC1-binding aptamer (**PEG-MUC1-MSN(palbo)**). These nanoparticles are designed to specifically target the human TNBC MDA-MB-231 cells, which overexpress the MUC1 receptor, and release palbociclib in the presence of glutathione (GSH) inside the cells, which reduces the disulfide bonds present in the capping ensemble. First, the first nanoparticle induces senescence, modifying the environment. Then, a second nanoparticle, loaded with the senolytic navitoclax and coated with a hexa-galactooligosaccharide (**Gal-MSN(Nav)**), releases the cargo, eliminating tumor senescent cells (Figure 8).^55^

The nanoparticles were tested *in vitro* and *in vivo*. The targeting capacity of **PEG-MUC1-MSN(palbo)** in MDA-MB-231 cells and the induction of senescence were confirmed by confocal microscopy and flow cytometry. Besides, the combined treatment of MDA-MB-231 cells resulted in an effective specific elimination of senescent cells (IC_50_ of 1.56 μM). Finally, the proposed therapeutic strategy was evaluated in the MDA-MB-231 tumor-bearing BALB/C nude mice. The concomitant treatment with **PEG-MUC1-MSN(palbo)** plus **Gal-MSN(Nav)** resulted in a significant reduction in tumor growth. These results confirmed a positive therapeutic effect using two sets of nanoparticles acting cooperatively. Interestingly, the protocol enhances the performance of the drugs. The encapsulation remarkably minimized undesired drug side effects, protecting animals from weight loss, observed with free palbociclib in monotherapy or combined with free navitoclax. It is also noteworthy that **Gal-MSN(Nav)** significantly reduces off-target effects and platelet toxicity significantly. Besides, compared to the systemic administration of palbociclib that favors metastasis, its encapsulation resulted in a decrease in metastasis in the lungs. Yet more, **PEG-MUC1-MSN(palbo)** plus **Gal-MSN(Nav)** resulted in the greatest decrease in metastasis compared to that of free drugs. Collectively, these findings demonstrated the efficacy of the nanoparticles that act cooperatively and “communicate” through stigmergy (a strategy in which systems communicate by modifying the environment).^55^ Enabling communication with nanoparticles is a field gaining interest, intending to expand the potential of nanotechnology in advanced applications to improve the way that we diagnose and treat diseases.^56^

**Figure 9:**
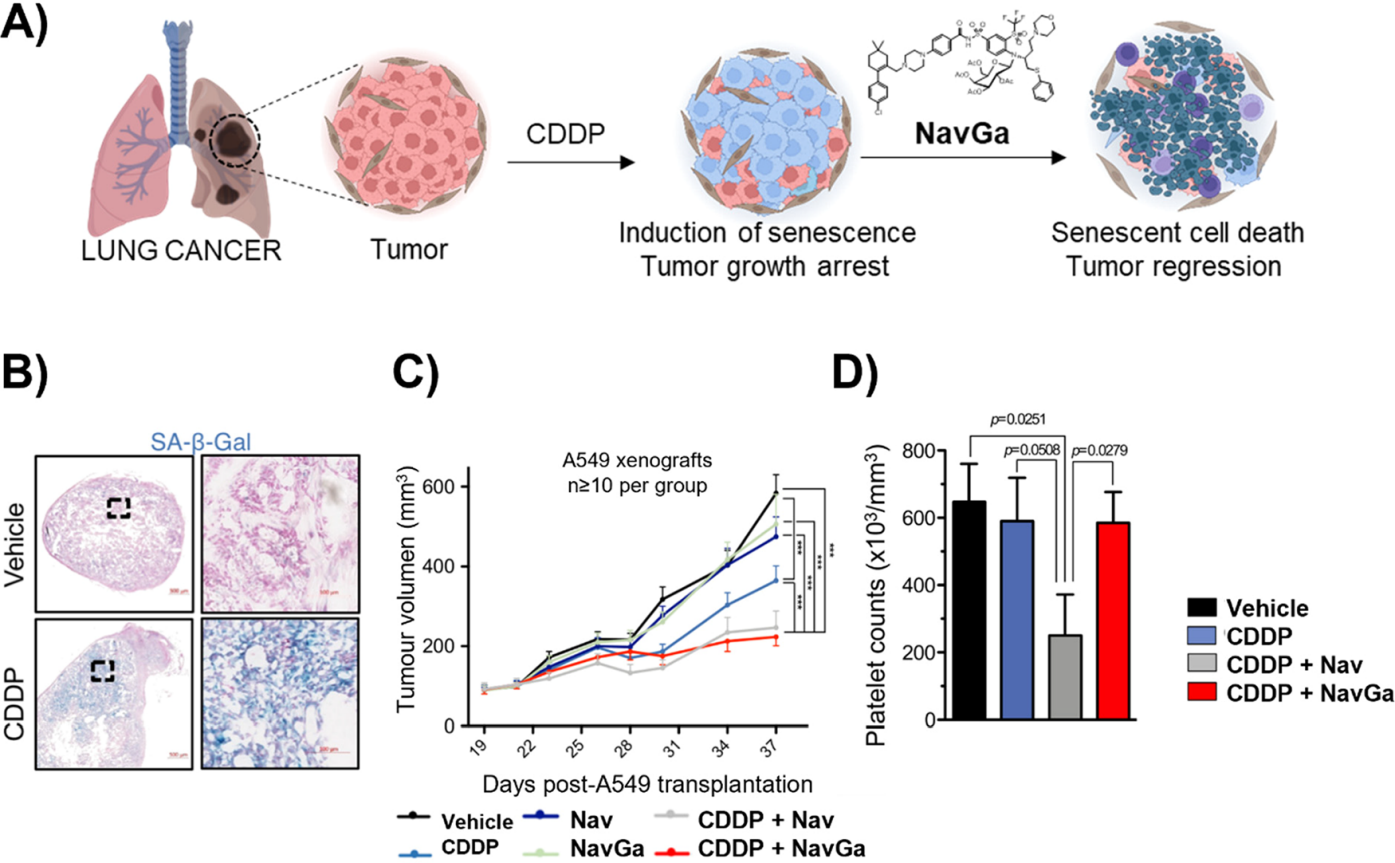
A) Schematic representation of the **NavGa** prodrug mechanism of action. B) Representative images of A549 xenografts stained for SA-β-Gal activity (in blue) following treatment with vehicle or cisplatin. C) A549 xenograft tumor volume in mice treated with cisplatin and navitoclax or **NavGa**. D) Platelet count after the treatment of wild-type C57BL/6J mice in each experimental condition. Adapted with permission from reference (4) with the Creative Commons CC BY license http://creativecommons.org/licenses/by/4.0/. Copyright 2020, the authors of the original publication.

As an alternative to the use of nanoparticles to selectively target senescent cells, we have also explored the design of prodrugs for enhanced senescence cell elimination.^4^ In this area, we prepared the prodrug **NavGa** that consists of a navitoclax molecule conjugated with an acetylated galactose unit. **NavGa** is activated by β-galactosidase (giving navitoclax) in a wide range of cell types (Figure 9A). The treatment of A549 lung carcinoma cells with cisplatin (CDDP) induced senescence and increased the sensitivity to navitoclax and **NavGa**, with low IC_50_ values (Figure 9B). Remarkably, the prodrug significantly reduces the toxicity of navitoclax to nonsenescent cells. A similar effect was observed for senescent SK-Mel-103 (induced with palbociclib), with an improved senolytic index in the case of **NavGa** over navitoclax, protecting nonsenescent cells. The navitoclax and **NavGa** prodrug decreased the tumor size *in vivo* in the lung adenocarcinoma mice model (A549 cells transplanted subcutaneously into immunodeficient (SCID) mice) and in non-small-cell lung cancer (C57BL/6J mice with a syngeneic luciferase-expressing KP lung adenocarcinoma cell line (L1475luc)) in combination with senescence-inducing therapies (Figure 9C). Remarkably, **NavGa** demonstrated a significant reduction in platelet toxicity compared to that of free navitoclax, as it did not lower platelet counts in treated mice or *ex vivo* in human blood samples due to the selective activation only in senescent cells (Figure 9D). This work suggests that galacto modification is a potential strategy to improve the senolytic specificity as well as the safety profile of current senolytic drugs.

We also validate the efficacy of the **NavGa** prodrug for TNBC therapy.^57^**NavGa** effectively eliminates senescent MDA-MB-231 cells (induced with palbociclib) with an improved senolytic index. The **NavGa** prodrug exhibited lower cytotoxic in nonsenescent MDA-MB-231 cells, thus demonstrating their selective activity in senescent cells. The effectivity of the combined treatment (palbociclib + **NavGa**) was also evaluated *in vivo* in the MDA-MB-231 orthotopic hTNBC balb/c mouse model. Tumor development was greatly reduced by the combined strategy with **NavGa** (and also with navitoclax), with strong cell death induction in senescent tumors as well as a significant decrease in the metastatic lung burden. These findings support the versatility of **NavGa** as a safer alternative for pro-senescent and targeted senolytic therapy.

**Figure 10:**
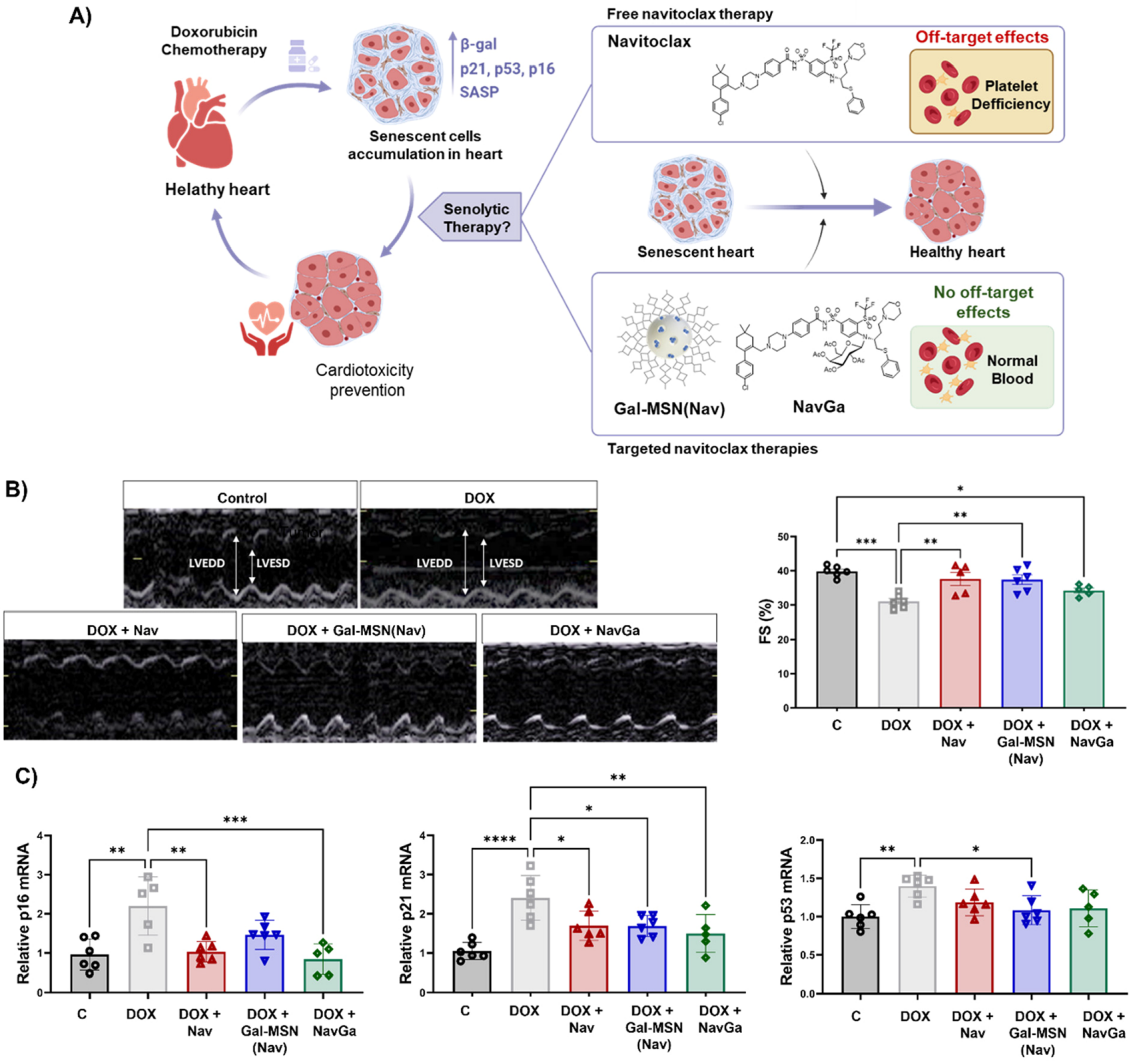
A) Representation of therapies based on navitoclax in a model of doxorubicin-induced cardiotoxicity. B) Representative echocardiographic analysis of mice under each experimental condition displaying changes in left ventricle (LV) systolic function and fractional shortening (FS) values obtained from animals at the experimental end-point (day 30). After injection with doxorubicin, a reduction in left ventricular (LV) contraction is observed and fractional shortening (FS), which is mitigated with senolytic treatment. C) Senescence markers p16, p21, and p53 mRNA expression in the hearts of mice under each experimental condition. Treatment with doxorubicin upregulates the expression of both markers in heart tissue; this indicated the senescent cell accumulation. This upregulation is reversed upon the administration of a senolytic treatment. Adapted with permission from reference (62). Copyright 2022, Elsevier.

Another interesting approach in which the targeted elimination of senescent cells can be exploited is the alleviation of doxorubicin-induced cardiotoxicity. Doxorubicin exposure severely affects the population of cardiac cells by inducing premature senescence.^58,59^ The accumulation of senescent cardiac cells is related to a decline in the regeneration capacity of the heart, thus causing long-term toxicity for patients.^60,61^ Based on that and taking advantage of our different targeted strategies (**Gal-MSN(Nav)** and **NavGa**), we evaluate the impact of senolysis to alleviate cardiac dysfunction associated with senescence (Figure 10A).^62^ First, the effectiveness of the senolytic therapy was analyzed in proliferating and doxorubicin-induced senescent cardiac myocytes. **Gal-MSN(Nav)** was nearly 4-fold more efficient than free navitoclax. **NavGa** exhibits superior and noteworthy protection on control cells even at high doses while maintaining a similar senolytic activity with respect to free navitoclax. We also validated our approach in female mice that were treated with either saline (control) or doxorubicin for 4 weeks, on days 1 and 4 every week (accumulative dose, 20 mg/kg) followed by senolytic treatments. At the end-point of doxorubicin treatment, while fractional shortening (FS) progressively decreased in mice treated with doxorubicin, a protective effect of the three formulations of navitoclax was evident in cardiac function (i.e., **Gal-MSN(Nav)**, **NavGa** and free navitoclax) (Figure 10B). Besides, after doxorubicin treatment, the expression of senescent markers *p16*, *p21*, and *p53* upregulated in cardiac tissue was decreased upon senolytic treatments (Figure 10C). The results demonstrated for the first time that the navitoclax systemic administration in various formulations proves effectiveness in eliminating senescent cells in the heart and correlates with the prevention of cardiac dysfunction in doxorubicin-induced cardiotoxicity. Although the therapeutic window is similar for the 3 senolytic formulations, in the case of **Gal-MSN(Nav)**, we observed results comparable to its counterparts even when administering a dose 40 times lower. In addition, targeted-senolysis offers the advantage of preventing the off-target effects associated with the administration of free navitoclax.

## 4 Conclusions and Prospects

This Account reports our recent research into the design of chemical strategies, such as the development of probes, controlled release nanodevices, and prodrugs, for the detection and elimination of cellular senescence and their *in vitro* and *in vivo* applications. We have reported galacto-conjugation of different molecular probes that has resulted in a suitable strategy for the effective real-time detection of senescence. We reported probes based on dyes displaying fluorescence at high wavelengths, some of them with two-photon capabilities showing higher tissue penetrance. Moreover, we have also described a renal clearable probe for the *in vivo* detection of β-galactosidase activity that can be correlated to the senescent burden in living animals. We have also explored the design of probes for the detection of lipofuscin and first demonstrated the use of nanoparticles for senescent cell detection. These and similar probes have great potential for the effective detection of the senescence, monitoring dysfunctional or damaged tissue in senescence-associated diseases, and tracking senolytic therapy. Along the same lines, the use of β-galactosidase activity-sensitive nanocarriers and prodrugs to increase the selectivity of currents drugs (for instance, navitoclax) has been demonstrated to eliminate senescent cells in different preclinical models. The main advantage of both targeted therapies (nanoparticles and prodrugs) is related to the reduction of off-target toxicity and side effects associated with current drugs, making them closer to clinical applications. Moreover, nanoparticles are a versatile tool easily adapted with different coatings and cargos, although more effort is needed to reach clinical applications. Considering the heterogeneous senescent phenotype, some innovative strategies are being investigated. Recently, the development of a new nanodevice responsive to the specific enzymatic activity of the senescent secretome has shown greater efficacy in the selective elimination of senescent cells.^63^

Overall, the data show that a new generation of probes and senolytics is emerging as an alternative for detecting, treating, and preventing senescence-associated disorders. However, the development of selective probes for real-time monitoring and the identification of more specific biomarkers remain challenging for more accurate detection and therapy. Technological advances and chemical strategies will be crucial in the near future for the further development of probes and senotherapies adaptable to specific senescence contexts.
